# Type-Based Morphological Reassessment of *Nola yoshinensis* and *N. kanshireiensis* (Lepidoptera, Nolidae), with Description of a Previously Misidentified New Species in Asia †

**DOI:** 10.3390/insects17101072

**Published:** 2026-10-16

**Authors:** Yeong-Bin Cha, Gyula M. László, Gábor Ronkay

**Affiliations:** 1 Forest Biodiversity Research Division, Korea National Arboretum, Pocheon 11186, Republic of Korea; 2 Department of Science, Natural History Museum, Cromwell Road, London SW7 5BD, UK; gyula.laszlo1@nhm.ac.uk; 3 Heterocera Press Ltd., Szt. István krt. 4, H-1137 Budapest, Hungary

**Keywords:** Oriental region, Palaearctic region, distribution, misidentification, type specimen, citizen science

## Abstract

The identities of *Nola yoshinensis* (Wileman & West) and *N. kanshireiensis* (Wileman & South) have long been confused in East Asia, leading to repeated misidentifications in Korea and Japan. In this study, we re-examined the primary type material of both nominal species to clarify their true identities. Our results show that neither *N. yoshinensis* nor *N. kanshireiensis* occurs in Korea. Instead, specimens previously identified under these names represent a distinct species. We therefore describe *Nola abscondita* Cha **sp. nov.** based on adult external and genital morphology. Illustrations of the adults and genital structures of the new species, as well as its distribution in Korea, Japan, and Taiwan, are provided. Furthermore, we integrated citizen science data from iNaturalist to supplement the distributional data of the treated taxa. Notably, these extralimital records reveal previously undocumented occurrences, revealing an extended range of these species in broader regions of Asia. The taxonomic implications of this reassessment are discussed, resulting in the introduction of new synonymies.

## Simple Summary

For a long time, a moth species occurring in East Asia was identified as *Nola yoshinensis* or *Nola kanshireiensis*. *Nola yoshinensis* was reported from Korea and Japan, while *Nola kanshireiensis* was believed to occur in Taiwan, Hong Kong, Korea, and Japan. By re-examining the original reference specimens of both species, we found that their true distributions are much more restricted. *Nola yoshinensis* occurs only in Japan, and *Nola kanshireiensis* is limited to Taiwan and Hong Kong, with neither occurring in Korea. Consequently, specimens from Korea, Japan, and Taiwan inconsistent with these verified distributional ranges represent a distinct, undescribed species, which is described here as new to science. Additionally, by analyzing community-contributed data from the citizen science platform iNaturalist, we provided new geographical records for these species. These online observations suggest that their actual ranges could be much wider than currently documented. This study demonstrates how re-examining well-known taxa through the combination of historical museum collections and modern citizen science can reveal hidden biodiversity and clarify species distributions.

## 1. Introduction

The genus *Nola* Leach, 1815 was established with the Palaearctic species *Phalaena cucullatella* Linnaeus, 1758 as the type species. This highly diverse noline genus is distributed across all continents except Antarctica. Asian representatives of *Nola* have been the focus of taxonomic investigation for more than a century, beginning with foundational works by authors such as Butler [1], Staudinger [2], and Hampson [3]. More recent contributions by Wileman and West [4], Inoue [5,6,7,8,9,10], Oh [11], László et al. [12], László et al. [13], Hu et al. [14], Cha et al. [15], and Yu and Hu [16], among others, have substantially advanced knowledge of the Asiatic fauna and expanded the number of recognized species to well over 200.

In East Asia, numerous studies have broadened our understanding of species diversity within the genus, with nearly 70 species currently documented [15,16]. Despite this long history of research, several taxonomic issues remain unresolved, particularly among morphologically similar species complexes. Members of the genus *Nola* are morphologically characterized by a small to medium body and three distinct raised scale-tufts in the forewing. In the male genitalia, the uncus is typically reduced, the valva is deeply cleft into a heavily sclerotized dorsal lobe and relatively membranous ventral lobe, and the harpe-clasper is well-developed. In the female genitalia, the corpus bursae is membranous, typically bearing one or two signa [17,18,19].

One of the most influential taxonomic treatments of Asian *Nola* was provided by Inoue [9], whose work has served as the primary identification source for the East Asian fauna. Numerous subsequent studies have relied on this treatment for species identification. However, as noted by Cha et al. [15], although Inoue [9] remains a fundamental monographic work on Asian *Nola*, several historical primary type specimens housed in European museums were not examined in the course of the preparation of the book, resulting in taxonomic uncertainties in later research. One of the misidentifications in Inoue [9] concerns *Nola yoshinensis* (Wileman & West, 1929), where our subsequent examinations confirmed that the specimen illustrated in Moths of Japan is not conspecific with the holotype of the species housed in the NHMUK.

In Korea, Oh [11] reviewed the genus *Nola* and reported 12 species, including *Nola yoshinensis*, following the species concept of Inoue [9]. This erroneous treatment was subsequently adopted by Kononenko and Han [20] in their synthesis of Korean noctuoid moths, which was also based on Oh’s unpublished doctoral thesis [21]. As a result, the incorrect concept of *N. yoshinensis* sensu Inoue became widely accepted in the East Asian Nolinae literature.

A further misinterpretation was proposed by Sasaki [22], who reassigned *N. yoshinensis* sensu Inoue as a synonym of *Nola kanshireiensis* (Wileman & South, 1916), a species described from Taiwan, despite the type locality of *N. yoshinensis* being Japan. However, this taxonomic change was not supported by illustrations or a detailed examination of genital morphology, and the rationale for the reassignment therefore remained unclear. Nevertheless, this treatment was subsequently followed in the Korean Noctuoidea checklist [23].

Recent studies have highlighted the importance of re-evaluating long-standing identifications within East Asian *Nola*. Cha et al. [15] demonstrated that misidentifications can conceal previously unrecognized diversity by revealing *Nola galliphaga* Cha et al., 2022 as a distinct species. In the present study, we re-examine the identities of *Nola yoshinensis* and *Nola kanshireiensis* based on their primary type material to clarify their true distributions in Asia. While *Nola yoshinensis* and *N. kanshireiensis* are confirmed from their respective type localities in Japan and Taiwan, examination of material from Korea, Japan, and Taiwan demonstrates that a third, distinct species occurs sympatrically with *N. yoshinensis* in Japan and with *N. kanshireiensis* in Japan and Taiwan, and represents the only species of this group occurring in Korea. This species is described here as *Nola abscondita* Cha **sp. nov.**

Through the re-examination of *N. yoshinensis* and *N. kanshireiensis*, we identified several junior synonyms. This led to the conspecific integration of multiple species and revealed that their actual geographical distributions are far more extensive than previously recognized.

Specifically, citizen science initiatives can provide highly reliable distributional data. Online platforms, most notably iNaturalist, host an extensive repository of photographic records contributed by community observers worldwide. These crowdsourced data have increasingly facilitated research into species ranges, spatial distributions, and novel taxonomic discoveries [24,25]. In the present study, we utilized iNaturalist records to supplement the distributional data for the taxa studied herein.

## 2. Materials and Methods

Specimens examined are preserved in the collection of the NHMUK and the private collection of the first author. The specimens in coll. Y-B. Cha were captured using tent light sheets illuminated by a mercury vapor lamp (220V/200W; Dongseong Co., Namyangju, Republic of Korea) and four blacklight lamps (18W; FL20SBL; Kumho Co., Seoul, Republic of Korea).

Images of INU adults were taken by a Tucsen Dhyana 400DC digital camera (Tucsen Photonics Co., Ltd., Fuzhou, China) attached to a Leica S6D stereomicroscope (Leica, Wetzlar, Germany), with dome illuminator Leica LED5000 HDI (Leica, Wetzlar, Germany). Genitalia were dissected and examined under a Leica S9E stereomicroscope (Leica, Wetzlar, Germany) or Leica EZ4 stereomicroscope (Leica, Wetzlar, Germany). Images of genitalia photographs were taken using a Tucsen Dhyana 400DC digital camera mounted on a Leica Z16 APO stereomicroscope (Leica, Wetzlar, Germany). Images of NHMUK adults were taken by a Nikon D90 digital camera (Nikon Corp., Tokyo, Japan) equipped with Nikkor AF Micro 60 mm lens (Nikon Corp., Tokyo, Japan). Genitalia dissected from NHMUK specimens were photographed using a Canon EOS 700D digital camera (Canon Inc., Tokyo, Japan) mounted on a Nikon SMZ 800 stereomicroscope (Nikon Corp., Tokyo, Japan) or by a Canon EOS 5DSR camera (Canon Inc., Tokyo, Japan) and MP-E 65 mm lens (Canon Inc., Tokyo, Japan) attached to a Kaiser RS 1 Repro stand (Kaiser Fototechnik GmbH & Co. KG, Buchen, Germany) equipped with a Stackshot system operated by Helicon Remote software (version 3.8.4 W) (Helicon Soft Ltd., Kharkiv, Ukraine) (NHMUK).

The holotype label data have been transcribed verbatim in quotation marks and with “/” denoting a different label.

## 3. Results

### 3.1. Taxonomic Account

Superfamily Noctuoidea Latreille, 1809

Family Nolidae Bruand, 1846

Subfamily Nolinae Hampson, 1894

***Nola* Leach** [26]: 135. Type species: *Phalaena Noctua palliola* [Denis and Schiffermüller], 1775 [junior subjective synonym of *Phalaena cucullatella* Linnaeus, 1758] by monotypy.

*Lira* Billberg [27]: 90. Type species: *Noctua palliola* [Denis and Schiffermüller, 1775]

*Chlamifera* Hübner [28]: 55. Type species: *Noctua palliola* [Denis and Schiffermüller, 1775]

*Roeselia* Hübner [29]: 397. Type species: *Phalaena cucullatella* Linnaeus, 1758

*Pisara* Walker [30]: 117. Type species: *Pisara opalina* Walker, 1862

*Necla* Walker [31]: 99. Type species: *Necla canioralis* Walker, 1863

*Automala* Walker [32]: 60. Type species: *Automala semidolosa* Walker, [1863] 1864

*Celama* Walker [33]: 500. Type species: *Celama liparisalis* Walker, 1865

*Aradrapha* Walker [34]: 1182. Type species: *Aradrapha partitalis* Walker [1866]

*Tribunta* Walker [34]: 1506. Type species: *Tribunta biguttalis* Walker, [1866]

*Lebena* Walker [35]: 1901. Type species: *Lebena trinotata* Walker, 1866

*Minnagara* Walker [35]: 1902. Type species: *Minnagara fasciata* Walker, 1866

*Argyrophyes* Grote [36]: 176. Type species: *Argyrophyes cilicoides* Grote, 1873

*Sorocostia* Rosenstock [37]: 435. Type species: *Eromene vetustella* Walker, 1866

*Epizeuctis* Meyrick [38]: 463. Type species: *Epizeuctis innocua* Butler, 1880

*Stenola* Möschler [39]: 119. Type species: *Stenola bistiga* Möschler, 1890

*Deltapterum* Hampson [3]: 138. Type species: *Deltapterum peguense* Hampson, 1894

*Neonola* Hampson [40]: 4. Type species: *Neonola mesosticta* Hampson, 1900

*Poliothripa* Hampson [41]: 312. Type species: *Poliothripa niphostena* Hampson, 1902

*Celamoides* van Eecke [42]: 120. Type species: *Celamoides pseudastigma* van Eecke, 1920

*Idiocyttara* Turner [43]: 35. Type species: *Sorocostia tornotis* Meyrick, 1888

#### 3.1.1. *Nola kanshireiensis* (Wileman & South, 1916)

***Celama kanshireiensis*** Wileman & South [44]: 179. Type-locality: [Taiwan] Formosa, Kanshirei. Holotype: female, in coll. NHMUK.

*Nola kanshireiensis* (Wileman & South): Poole [45]: 697; László & Sterling [46]: 43.

⁠

*Nola sakishimana* Inoue, 2001, **syn. nov.**

*Nola sakishimana* Inoue [47]: 226. Type-locality: Japan, Ishigakijima, Maesato. Holotype: female, in coll. NHMUK.

*„Nola” sakishimana* Inoue: László, Ronkay and Witt [12]: 63.

*Nola sakishimana* Inoue: Sasaki [22]: 177.

⁠

*Nola spinivesica* Holloway, 2003, **syn. nov.**

*Nola spinivesica* Holloway [18]: 58. Type-locality: (Malaysia) Sarawak, Gunong Mulu National Park. Holotype: male, in coll: NHMUK.

*Nola spinivesica* Holloway: Joshi et al. [48]: 276; Joshi et al. [49]: 76.

⁠

**Holotype** of *Celama kanshireiensis* Wileman & South, 1916: f#, circular label with red margin “Type” / [with printed] “Kanshirei, Formosa. 1,000 ft. 9. IV. 1908. A. E., Wileman” / [with handwritten] “Celama kanshireiensis sp. n. Type ♀” / [with printed] “Wileman Coll. B. M. 1929-261.” / [with handwritten] “Nola major Nol 5. R.S” / [with handwritten in red] “1279F”, in coll. NHMUK.

**Paratypes** of *Nola sakishimana* Inoue, 2001: 1 m#, “JAPAN: Okinawa, Iriomote Is., Urauti, 22-Mar-1995, T. Mano leg.” / “Inoue coll. BM 2008-114” / [yellow label] “PARATYPE *Nola sakishimana* Inoue, 2001”, genitalia slide No. [Inoue] 16688, QR code label with unique id. NHMUK 014203521; 1 f#, “JAPAN: Okinawa, Iriomote Is., Hunaura, 24-Jul-1995, T. Mano leg.” / “Inoue coll. BM 2008-114” / [yellow label] “PARATYPE *Nola sakishimana* Inoue, 2001”, genitalia slide No. [Inoue] 16689, QR code label with unique id. NHMUK 014203522, in coll. NHMUK.

**Holotype** of *Nola spinivesica* Holloway, 2003: m#, circular label with red margin “HOLOTYPE” / “SARAWAK: Gunong Mulu Nat. Park, R.G.S. Exped. 1977-8, J.D. Holloway et al., B.M. 1978-206” / “Site 10. February, Camp 1, Mulu 160 m. 386470, Mixed dipt. for. Ac.-understorey.” / “Figured specimen Moths of Borneo Part 18” / “Holotype Nola spinivesica Holloway” / [blue label] “Noctuidae genitalia slide No. 17517 m#”, in coll. NHMUK.

**Figure 1:**
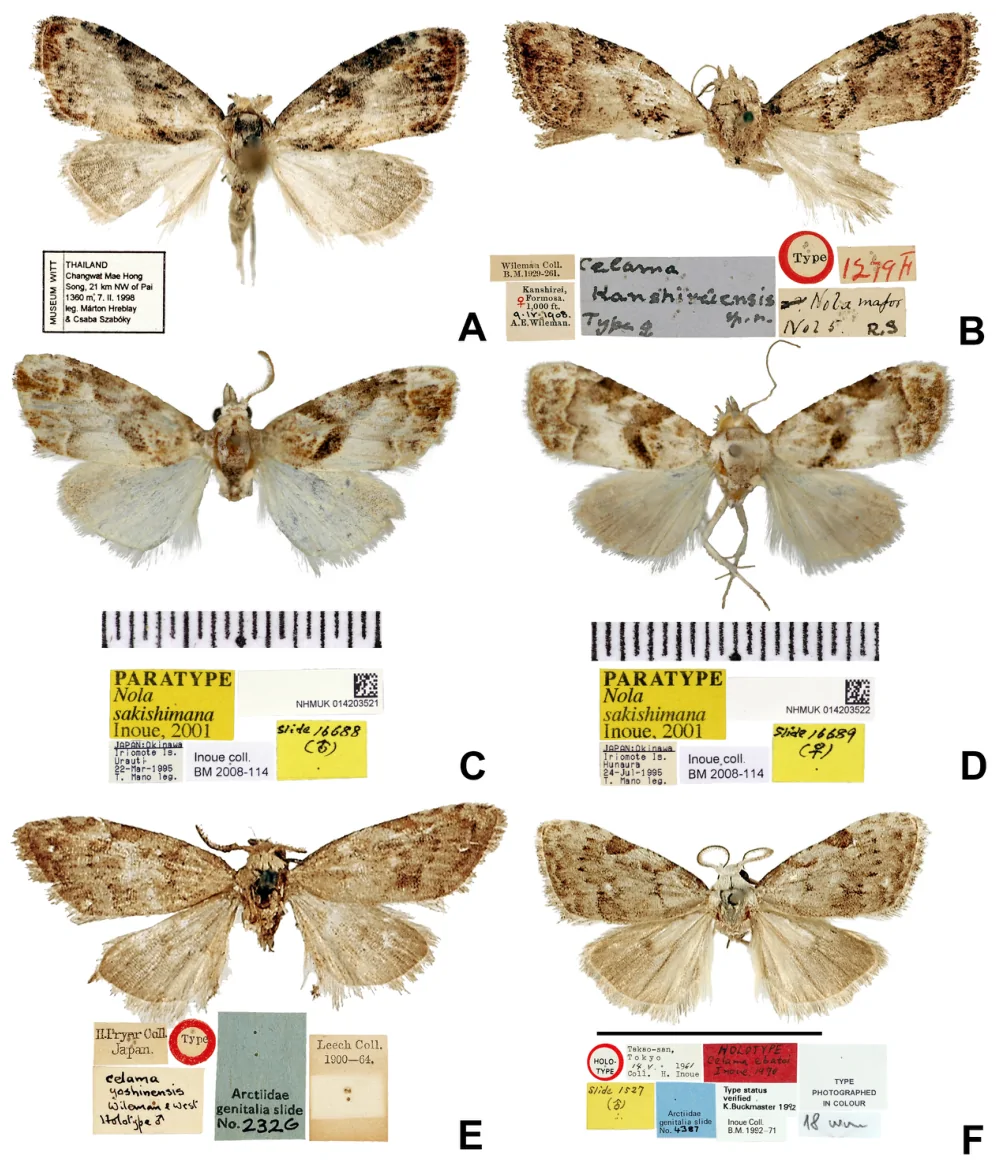
Type and additional material of *Nola kanshireiensis* (**A**–**D**) and *N. yoshinensis* (**E**,**F**). (**A**) male, Thailand, in coll. MWM/ZSM; (**B**) Holotype with labels, female, in coll. NHMUK; (**C**) Paratype of *N. sakishimana* **syn. nov.** with labels, male, in coll. NHMUK; (**D**) Paratype of *N. sakishimana* **syn. nov.** with labels, female, in coll. NHMUK; (**E**) Holotype with labels, male, in coll. NHMUK; (**F**) Holotype of *N. ebatoi* **syn. nov.** with labels, male, in coll. NHMUK.

**Redescription.** Adult (Figure 1A–D). Antenna bipectinate with short rami in male, filiform in female. Head and thorax greyish-cream. Ground color of forewing greyish-cream; basal area with mottled brown spots; antemedial line strongly curved, medially angled, rather broad, often diffuse or accompanied by dark brown elongate patches. Medial area variably irrorated with dark brown scales, costa with large blackish-brown elongate patch; medial line deleted. Postmedial line diffuse, dark brown, zigzagged, often poorly visible; subterminal line sinuous, interrupted, consisting of blackish spots; subterminal area red-brown; terminal line beige. Cilia grey, irrorated with blackish scales. Ground color of hindwing pale grey with dense brownish-grey irroration distally; grey discal spot pale, dash-like (hindwings of holotype specimen are damaged). Abdomen greyish-cream (missing in the holotype specimen).

**Figure 2:**
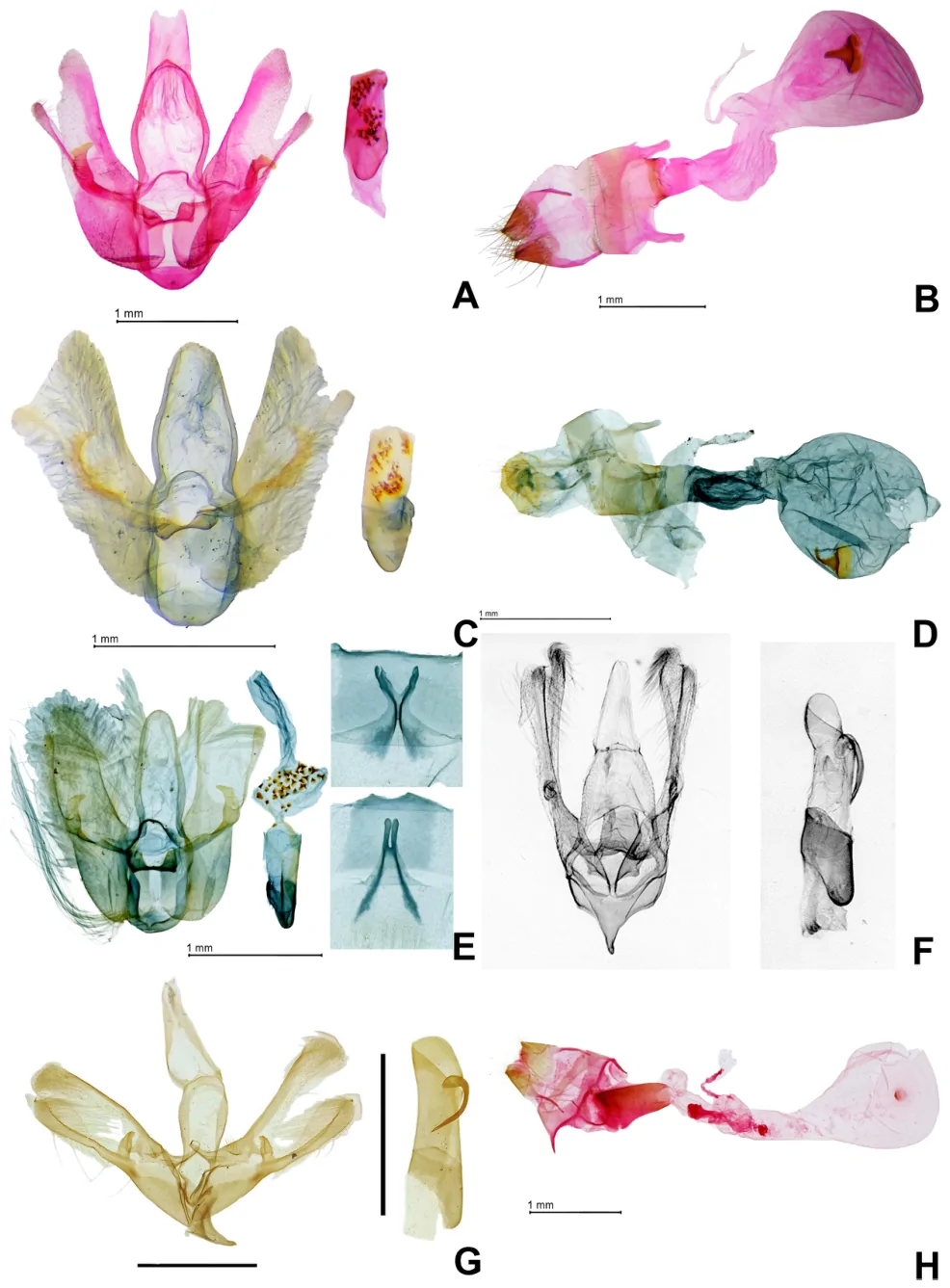
Type and additional genitalia of *Nola kanshireiensis* (**A**–**E**) and *N. yoshinensis* (**F**–**H**) (all in coll. NHMUK). (**A**) male, Hong Kong, gen. slide No. LGN 2875; (**B**) female, Hong Kong, gen. slide No. LGN 2908; (**C**) Paratype of *N. sakishimana* **syn. nov.**, male, Japan, Inoue gen. slide No. 16688; (**D**) Paratype of *N. sakishimana* **syn. nov.**, female, Japan, Inoue gen. slide No. 16689; (**E**) Holotype of *N. spinivesica* **syn. nov.**, male, Sarawak, BM Noctuidae genitalia slide No. 17517, with 8th segment (up: tergite, down: sternite); (**F**) Holotype, male, Japan, BM Arctiidae genitalia slide No. 2326; (**G**) Holotype of *N. ebatoi* **syn. nov.**, male, Japan, BM Arctiidae genitalia slide No. 4387; (**H**) *N. ebatoi* **syn. nov.**, female, Japan, gen. slide No. NHMUK014334334.

Male genitalia (Figure 2A,C,E). Uncus entirely reduced, subscaphium membranous with weakly sclerotized, thin longitudinal bars. Tegumen very narrow, elongate. Valva bilobated; dorsal lobe elongate-triangular with weakly sclerotized, relatively broad costa and rounded apex; ventral lobe originating postmedially, short and narrow digitiform with sclerotized ventral margin and rounded apex; harpe rather broad, distally dilated with slightly produced rounded apex pointing inward. Juxta short bowl-shaped. Sacculus rather stout, short. Vinculum short, broad U-shaped. Aedeagus short and thick with short, rounded caecum and unmodified distal end; vesica with extensive cornuti field consisting of short but robust, somewhat thumbtack-shaped spines.

Female genitalia (Figure 2B,D). Papilla analis short, trapezoidal. Apophysis posterioris medium-long, rod-shaped; apophysis anterioris broader and ca. 50% shorter than apophysis posterioris. Eighth tergite short, ribbon-like with more or less parallel margins. Ostium bursae well-sclerotized, rather broad, rectangular. Ductus bursae relatively short, longitudinally striate, ventrally bulged. Cervix bursae slightly protruding, rounded, membranous, ductus seminalis thin. Corpus bursae ovoid, with a large thorn-shaped signum bursae.

**Distribution.** Japan (Ryukyus), China (Taiwan, Hong Kong), Thailand (Mae Hong Song), India, Malaysia (Sarawak), Indonesia (Bali) [12,18,22,46,48,49].

**Remark.** The female holotype of *N. kanshireiensis* lacks the abdomen; however, owing to the species’ characteristic wing pattern and the absence of sexual dimorphism, the genital morphology of both sexes were reliably clarified based on specimens from Hong Kong [46].

Since no consistent diagnostic differences could be identified between this species and *N. sakishimana* Inoue, 2001 (described from Japan) or *N. spinivesica* Holloway, 2003 (known from Borneo, Bali, and India), these latter two taxa are here considered junior synonyms of *N. kanshireiensis*.

#### 3.1.2. *Nola yoshinensis* (Wileman & West, 1929)

***Celama yoshinensis*** Wileman & West [4]: 187. Type locality: Japan.

*Celama yoshinensis* Wileman & West: Matsumura [50]: 92

*Nola yoshinensis* (Wileman & West): Poole [45]: 702.

⁠

*Nola ebatoi* (Inoue, 1970), **syn. nov.**

*Celama ebatoi* Inoue [7]: 2. Type locality: Japan, Tokyo, Takao-san.

*Nola ebatoi* (Inoue): Inoue [9]: 662; Sasaki [22]: 174.

**Holotype** of *Celama yoshinesis* Wileman & West, 1929: m#, circular label with red margin “Type” / [with printed] “H. Pryer Coll. Japan.” / [with printed] “Leech Coll. 1900-64.” / [with handwritten] “Celama yoshinensis Wileman & West Holotype ♂” / “Arctiidae genitalia slide No. 2326”, in coll. NHMUK.

**Holotype** of *Celama ebatoi* Inoue, 1970: m#, circular label with red margin “HOLOTYPE” / [with partially handwritten] “Takao-san, Tokyo 14.v.1961 Coll. H. Inoue” / [red label, with handwritten] “HOLOTYPE Celama ebatoi Inoue, 1970” / [yellow label, with handwritten] “Slide 1527 (m#)” / [printed] “Inoue Coll. B.M. 1992-71” / “Type status verified K. Buckmaster 1992” / “Type photographed in colour” / “Arctiidae genitalia slide No. 4387”, in coll. NHMUK.

Additional material examined. 1 f#, Japan, Bushi, Iruma, Saitama Pref., 20. IV. 1977, H. Inoue leg., NHMUK016480178, gen slide No.: NHMUK014334334 (coll. NHMUK).

**Redescription.** Adult (Figure 1E,F). Male antenna bipectinated with short rami. Head and thorax whitish-grey to pale brown. Ground color of forewing pale brownish-grey. Antemedial line thin, diffuse, arched, slightly dentate medially; postmedial line double, rather broad, gently S-shaped, inner line diffuse, shadow-like, outer line defined by dark brown dots; medial area with prominent rust-brown semi-circular to trapezoidal costal patch. Subterminal line strongly undulate, diffuse; terminal line dark brown; cilia greyish-brown. Hindwing ground color pale brownish-grey, slightly darkened toward apex, discal spot diffuse, ovoid; cilia pale brownish-grey.

Male genitalia (Figure 2F,G). Uncus entirely reduced, with weakly sclerotized subscaphium. Tegumen moderately elongate, narrow. Valva deeply cleft bilobate; dorsal lobe spatulate with setose rounded apex, ca. 20% longer than ventral lobe, costal margin well-sclerotized, with a broad, weakly sclerotized pointed field near base with a small hairy editum. Ventral valval lobe tongue-shaped, ventral margin well sclerotized, apex with tiny spine. Harpe relatively broad basally, tapered distad, apex pointed distal half slightly arched. Sacculus short and narrow, weakly sclerotized. Juxta inverse-triangular shield-shaped with short anterolateral processes. Vinculum narrow V-shaped, apically pointed. Aedeagus short, moderately thick, caecum short, rounded, carina with large, strongly hooked, acute cornutus with tip pointing proximally, length almost 1/3 the length of aedeagus.

Female genitalia (Figure 2H). Papilla analis short, trapezoidal. Apophysis posterioris medium-long, rod-shaped; apophysis anterioris half as long as apophysis posterioris. Eighth tergite short, ribbon-like with more or less parallel margins. Ostium bursae well-sclerotized, rather thin, circular. Antrum funnel-like, heavily sclerotized, as long as membranous tubular section of ductus bursae with a rounded, membranous lateral protrusion at proximal end of antrum. Cervix bursae protruding, membranous, ductus seminalis thin. Corpus bursae with tubular distal section and ovoid proximal section with a small, ridge-like signum bursae.

**Distribution.** Japan [4].

**Remark.** Both *N. yoshinensis* and its junior synonym *N. ebatoi* were described from Japan. Subsequently, *N. ebatoi* was reported from Korea by Oh [11] and Kononenko and Han [20], however it was based on a misidentification. In addition, Beljaev et al. [51]: 403 reported *N. ebatoi* from the southern Amur region based on the same taxonomic concept that included the Korean records. Because the Korean records are demonstrated herein to be based on misidentifications, the identity of the material from the southern Amur region also requires confirmation. Therefore, in the present study, we follow the taxonomic concept of *N. ebatoi* adopted by Inoue [9] and Sasaki [22]. The true distribution of this species requires further investigation.

#### 3.1.3. *Nola abscondita* Cha **sp. nov.**

Zoobank: urn:lsid:zoobank.org:act:6FF4D1F8-468F-4452-80C2-D3F26442D72E

*Nola yoshinensis* (Wileman & West, 1929) (*sensu* Inoue): Inoue [9]: 663; Oh [11]: 129; Kononenko and Han [20]: 67.

*Nola kanshireiensis* (Wileman & South, 1916) (*sensu* Sasaki) Sasaki [22]: 175; Kim et al. [23]: 141.

⁠

**Holotype.** m#, Sang-ri 633-3, Yeoncheon-eup, Yeoncheon-gun (N38°07′59.3″, E127°06′56.3″), 12. VI. 2019 (Ko J.H., Kim H.), genitalia slide No. INU-11834, in coll. INU. **Paratypes.** (87m#, 82f#) **[Korea]** [CB] 1m#, Mt. Weolaksan, 31. V. 1986 (Paik W.H.), genitalia slide No. INU-11556; 1m#, Danyang, 28. VII. 1995 (Paik M.K.); 1m#, Susan 1-ri, 7. V. 1999 (Sohn J.C.), genitalia slide No. INU-11514; 1m#, Cheongpung resort, 9. V. 2008 (Bae Y.S., Ju Y.D., Chae D.Y.), genitalia slide No. INU-11632; [CN] 1f#, Sindu-ri 969, Wonbuk-myeon, Taean-gun (N36°50′11.02″, E126°11′42.46″), 9. IX. 2019 (Jang C.M.); [GB] 1m#, 1f#, Ulleung Is., Ulleung-gun, 19. VI. 2006 (Bae Y.S., Cha D.Y.), genitalia slide Nos. INU-11656, -11658; 5f#, Yaksu park, Ulleung-gun, 22. VI. 2007 (Bae et al.), genitalia slide Nos. INU-11644, -11645, -11646, -11647, -11648; 1m#, Mt. Chunchuksan, Uljin-gun, 16. IX. 2014 (Ju Y.D., Na S.M., Kim K.W.), genitalia slide No. INU-11670; 2m#, Buljeong-dong 148, Mungyeong-si (N36°37′59.7″, E128°08′48.4″), 25. IX. 2019 (Ko J.H., Cha Y.B., Kim H.); 1f#, Ugok-ri, Sanbuk-myeon, Mungyeong-si (N36°41′53″, E128°14′2″), 13. VII. 2020 (Bae et al.), genitalia slide No. INU-11640; 1f#, Gyeryongsan National Park, 13. VII. 2021 (Oh J.I.); [GG] 1f#, Seoul, 17. VII. 1971 (Lee S.M.); 1f#, Suweon, 12. VI. 1974 (Park K.T.), genitalia slide No. INU-11651; 2f#, Suweon, 23. VII. 1974 (Park K.T.); 1f#, Suweon, 24. VII. 1974 (Park K.T.), genitalia slide No. INU-11633; 1f#, Suweon, 23. VIII. 1974 (Park K.T.), genitalia slide No. SH-105; 1f#, Suweon. 28. VIII. 1974 (Park K.T.); 2m#, Suweon, 10. IX. 1974 (Lee Y.I.), genitalia slide Nos. INU-11527, -11547; 1f#, Suweon, 23. VII. 1975 (Park K.T.); 2m#, Suweon, 13. VII. 1975 (Park K.T.), genitalia slide Nos. SH-103, INU-11642; 1m#, Mt. Cheonmasan, 28. V. 1976 (Lee S.M.); 7m#, 1f#, Suweon, 16. IX. 1976 (Park K.T.), genitalia slide Nos. SH-42, -144, INU-11634, -11639, -11652, -11667; 2f#, Suweon, 20. VII. 1976 (Hwang C.Y.); 1f#, Gwangneung, 8. VI. 1977 (Paik J.C.), genitalia slide No. SH-43; 1m#, 1f#, Gwangreung, 10. VII. 1982 (Park K.T.), genitalia slide No. SH-106; 1m#, Suweon, 22. VIII. 1982 (Ryu C. H.), genitalia slide No. INU-11636; 1m#, 1f#, Cheongryang-ri, 5. IX. 1982 (Won G.J.), genitalia slide No. INU-11544; 1m#, Suweon, 14. IX. 1982 (Ryu C.H.), genitalia slide No. INU-11641; 1m#, Suweon, 14. IX. 1982 (Im D.J.); 1m#, Suweon, 16. IX. 1982 (Im D.J.); 1m#, Suweon, 17. IV. 1983 (An S.B.), genitalia slide No. INU-11637; 1f#, Cheongryang-ri, 5. VI. 1985 (Weon K.J.); 1f#, Mt. Chonggyesan, 4. VI. 1986 (Ahn S.B.), genitalia slide No. SH-44; 1f#, Mt. Cheonggyesan, 4. VI. 1986 (Ahn S.B.), genitalia slide No. INU-11643; 1f#, Gwangreung, 4. 4. VIII. 1988 (Park K.T.); 1f#, Suweon, 6. X. 1988 (Ahn S.B.); 1m#, Mt. Yumyeongsan, 17. VI. 1990 (Oh S.H., Choi H.Y.); 1f#, Mt. Myeongjisan, 14. IX. 1992 (Park K.T., Byun B.K.), genitalia slide No. INU-11526; 1f#, Yongyudo Is., Incheon, 28. VIII. 1997 (Paek M.K., Ahn N.H.), genitalia slide No. INU-11669; 3m#, 1f#, Seonjaedo Is., 23. V. 1999 (Paek, Kim, Kim), genitalia slide Nos. INU-9120, -11624, -11663, -11664; 2m#, Yeongheungdo Is., 29. V. 1999 (Bae, Paek, Kim, Kim), genitalia slide No. INU-11659, -11660; 3f#, Changdeokgung palace, Seoul, 30. VIII. 1999 (Paek, Lee, Kim), genitalia slide No. INU-11661, -11662; 1f#, Mueuido Is., Incheon, 18. IX. 2004 (Oh, Kim, Song), genitalia slide No. INU-11657; 1f#, Sanasa valley, Yangpyeong-gun, 22. VIII. 2013 (Ju, Kim, Park); 1m#, Godeok waterfront eco park, 16. V. 2017 (Na S.M., Kim H.K.), genitalia slide No. INU-11627; 2m#, 1f#, Bamseom Is., Hangang river, Seoul, 18. VII. 2019 (Bae Y.S., Lee T.G., Jang C.M.); 1f#, Bangi-dong Ecological Landscape Preservation Area, Seoul (N37°30′41.6″, E127°08′23.0″), 10. VI. 2020 (Cha Y.B., Jang C.M.), genitalia slide No. INU-11619; [GN] 2m#, 1f#, Gwunbin reservoir, Hapcheon, 13. IX. 2017 (Bae et al.), genitalia slide Nos. INU-11631, -11650; [GW] 1m#, Chuncheon, 21. V. 1983 (Park K.T.), genitalia slide No. SH-346; 1m#, Chuncheon, 27. V. 1984 (Park K.T.); 2m#, Experiment forest, Hongcheon, 5. IX. 1986 (Park K.T.), genitalia slide Nos. INU-11635, -11665; 3m#, 1f#, Experiment forest, Hongcheon, 10. VI. 1988 (Park K.T.), genitalia slide Nos. SH-104, INU-11517; 1f#, Pyeongchang, 24. VI. 1988 (Oh S.H.), genitalia slide No. INU-11654; 2f#, Chuncheon, 18. VII. 1989 (Park K.T.), genitalia slide No. INU-11557; 1m#, Chuncheon, 28. V. 1990 (Park K.T.), genitalia slide No. SH-158; 1m#, 2f#, Chuncheon, 30. VIII. 1990 (N37°56′, E127°44′), genitalia slide Nos. INU-11583, -11653, -11666; 1f#, Oweol-ri, Chuncheon, 5. V. 1994 (Park K.T.); 1m#, Chuncheon, 21. VII. 1992 (Park K.T., Byun B.K.), genitalia slide No. INU-11524; 14m#, 2f#, Choogok-ri, Chuncheon, 2. IX. 1996 (Lee H.K., Lee J.S.), genitalia slide Nos. INU-9108, -11515, -11516, -11521, 11522, -11560, -11561, -11630, -11673, -11674; 1f#, Mt. Taehwasan, Yeongweol-gun, 14. VII. 1998 (Lee J.S., Jung J.U.), genitalia slide No. INU-11513; 5f#, Temp. Baekdamsa, Mt. Seolaksan, Inje-gun, 4. VII. 2002 (Bae, Oh, Choi, Song), genitalia slide Nos. INU-12620, -11621, -11622, -11623, -11628; 1f#, Temp. Baekdamsa, Mt. Seolaksan, Inje-gun, 9. VII. 2003 (Bae, Oh, Choi, Song), genitalia slide No. INU-11625; 1f#, Mt. Baekwunsan, Wonju-si, 8. IX. 2011 (Qi M.J., Lee H.J., Na S.M.), genitalia slide No. INU-11649; 1m#, Broad-leaved tree, Wonju-si (N37°21′58.82″, E127°50′27.03″), 7. VII. 2016 (Na S.M., Lee D.J.); 1m#, Malgeunmul pension, Mt. Taegisan, Hoeongseong-gun (N37°35′44.4″, E128°14′37.6″), 9. VIII. 2018 (Bae Y.S., Lee D.J., Lee T.G., Cha Y.B., Heppner J.B.); 1m#, 1f#, Mt. Taegisan, Hoeongseong-gun, (N37°35′56.3″, E128°14′53.0″), 10. VIII. 2018 (Bae Y.S., Lee D.J., Lee T.G., Cha Y.B., Heppner J.B.), genitalia slide No. INU-9147; 2m#, 3f#, Panun-ri 1167, Jucheon-myeon, Yeongwol-gun (N37°12′59.44″, E128°20′08.06″); 1f#, Bangnim-ri 80-1, Bangnim-myeon, Pyeongchang-gun (N37°25′45.3″, E128°25′40.1″), 20. IX. 2018 (Ko J.H., Jang C.M.); 4m#, 2f#, Hanbando wetland, Yeongweol-gun (N37°12′59.44″, E128°20′08.06″); 4f#, Hanbando wetland, Yeongweol-gun (N37°12′59.44″, E128°20′08.06″); 21. V. 2019 (Lee T.G., Jang C.M.), genitalia slide No. INU-11837; 1m#, Sangno-ri 863, Dongsong-eup, Cheolwon-gun (N38°09′34.2″, E127°11′31.6″), 3. VI. 2019 (Ko J.H., Jang C.M); 2f#, Tongchon-ri 277-1, Hwacheon-myeon, Hwacheon-gun (N38°08′44.9″, E127°47′09.1″), 19. VI. 2019 (Ko J.H., Jang C.M.); 1f#, San 165-13, Omi-ri, Bangsan-myeon, Yanggu-gun (N38°10′40.5″, E127°55′24.2″); 1m#, Hanbando wetland, Yeongwol-gun (N37°13′25.29″, E128°19′33.5″), 23. VIII. 2019 (Bae Y.S., Jang C.M.); [JB] 3m#, 3f#, Mt. Daedunsan, 22. V. 1992 (Park K.T.), genitalia slide Nos. INU-11523, -11525, -11638; 3m#, 1f#, Hamyeol, Iksan-si (N36°4′31.36″, E126°54′44.05″), 19. V. 2017 (Na S.M.) genitalia slide Nos. INU-9107, -11626, -11629, -11655; [JN] 1f#, Mt. Jirisan, 14. VII. 1976 (Ha Y.Y.), genitalia slide No. INU-11668; 1m#, Mt. Baekwunsan, Gwangyang-si, 21. VIII. 1991 (Ahn S.B.), genitalia slide No. SH-365 (coll. Y.B. Cha). [GB] 1m#, Seodalryeong, Seo-myeon, Ulleungdo Is. (N37°30′30.57″, E130°49′07.4″), genitalia slide No. INU-11826; [GN] 1m#, Mt. Ganwolsan, Sangbuk-myeon (N35°33′13.24″, E129°03′54.70″), 31. V. 2007 (Lee B.W.), genitalia slide No. INU-11769; [GW] 1f#, Choogok-ri, Chuncheon, 2. IX. 1996 (Lee H.K., Lee J.S.), genitalia slide No. CIS Noc. 313; [JB] 1m#, 2f#, Udong-ri, Boan-myeon, Buan (N35°37′26.7″, E126°38′25.5″), 25. IX. 2013 (Lee B.W., Oh S.M.) (coll. KNAE). **[Japan]** 3m#, 1f#, Sakurabuchi, Shinshiro, Aichi Pref., 17–18. V. 1958 (Inoue H.), genitalia slide Nos Inoue 3414 ♂, 3470 ♂, 3415 ♀; 5m#, 1 f#, same site and collector, 4.x.1959; 3m#, Shinshiro, Aichi Pref., 9.viii.1962, H. Inoue, genitalia slide No. Inoue 3471; 2 m#, Numazu, Shizuoka, 17.v.1952, T. Haruta; 1 m#, Onyu, Obama City, Fukui Pref., 25.viii.1951, I. Izaki, genitalia slide No. Inoue 3436; 1m#, Onoaida, Yakushima, 23.iii.1972, T. Watanabe; 1f#, same site and collector, 14.vi.1972; 1f#, Kusukawa, Yakushima, 25.x.1971, T. Watanabe; 5f#, Kurio (Otaki), Yakushima, 29.x.1971, T. Watanabe; 1f#, Tokusima C., Kuramoto, 5.ix.1963, Coll. Y. Nagai; 1f#, same site and collector, 9.ix.1963 (coll. NHMUK); 1m#, 3f#, Makiosan, Osaka, Honshu, 17. IX. 1987 (Bae Y.S.), genitalia slide Nos. INU-12189, -12200, -12201 (coll. Y.B. Cha). **[Taiwan]** 2m#, Hwa-Lung, Wu-Cheng, Yu-Chi, Nantou H. (Alt.: 600 m), 18–19. XI. 1997 (Bae Y.S. & Park K.T.), genitalia slide Nos. INU-12209, 12210 (coll. Y.B. Cha); 1m#, Taipei, Wulai, 10.iv.1972, M. Owada, genitalia slide No. Inoue 12218; 1m#, Nantow, Rushan-Unchen, 19-21.iii.1972, M. Owada; 1f#, Taipei, Wulai, 17.vii.1964, H. Inoue, genitalia slide No. Inoue 12219; 1f#, Kuantunchi, Nan Tow, 29.x.1972, S. Yamane; 1f#, Hualien Hsien, Losao, 1110m, 2.iv.1984, A. Kawabe leg.; 1f#, Hualien Hsien, Hingyeh Spa, 200m, 29-30.iii.1984, S. Sugi leg., NHMUK 016480176, gen slide No.: NHMUK 014334333 (coll. NHMUK).

**Diagnosis.** *Nola abscondita* **sp. nov.** is readily distinguished from *N. yoshinensis* by the considerably darker coloration (dark brownish-grey instead of whitish-grey to pale brown) of the head, thorax and abdomen, as well as the fore- and hindwing, the more contrasting and more gently curved and not dentate antemedial line, the substantially smaller and less prominent mediocostal patch of the forewing and the noticeably smaller discal spot of the hindwing.

In the male genitalia, the new species differs from *N. yoshinensis* in having a markedly deeper medial cleft between the dorsal and ventral valval lobes, a slightly broader apex of the dorsal lobe, a substantially narrower ventral lobe, and a distinctly narrower, longer, and more acutely pointed harpe positioned closer to the valval base. In addition, the new species possesses a shorter, broadly rounded vinculum, in contrast to the pointed vinculum of *N. yoshinensis*. The most striking distinction between the two species lies in the structure of the cornutus vesicae: *N. abscondita* **sp. nov.** bears a basally robust, distally tapering, slightly curved, apically rounded, and comparatively short sub-triangular cornutus, whereas the allied species exhibits a very long, slender, strongly hooked, and sharply pointed cornutus.

The female genitalia of the new species are reminiscent of those of *N. aerugula* (Hübner), but differ in the shorter apophysis anterioris, the longer and broader sclerotized distal section of the ductus bursae, the absence of a membranous tubular portion of the ductus bursae, and the more extensively swollen and conspicuously rugose cervix bursae. The signum bursae of *N. abscondita* **sp. nov.** bears a more developed basal plate than that of *N. aerugula*, and possesses a short, narrow, apically rounded projection that is absent in the latter species.

**Figure 3:**
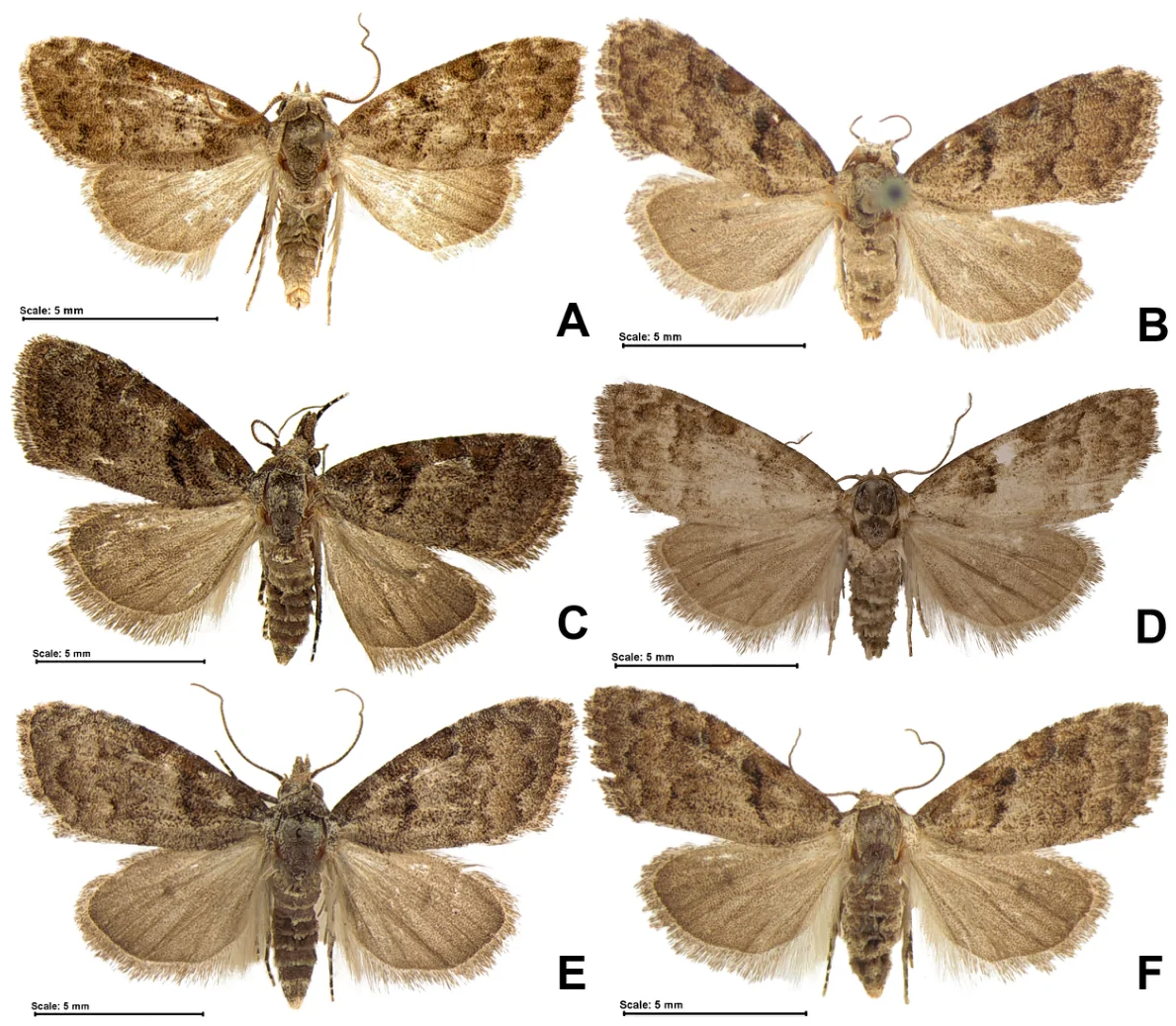
Adults of *Nola abscondita* Cha **sp. nov.** from Korea (all in coll. Y.B. Cha). (**A**) Holotype, male, INU-11834; (**B**) Paratype, female, INU-11666; (**C**) Paratype, male, INU-11627; (**D**) Paratype, female, INU-12318; (**E**) Paratype, male, INU-11629; (**F**) Paratype, female, INU-11669.

**Figure 4:**
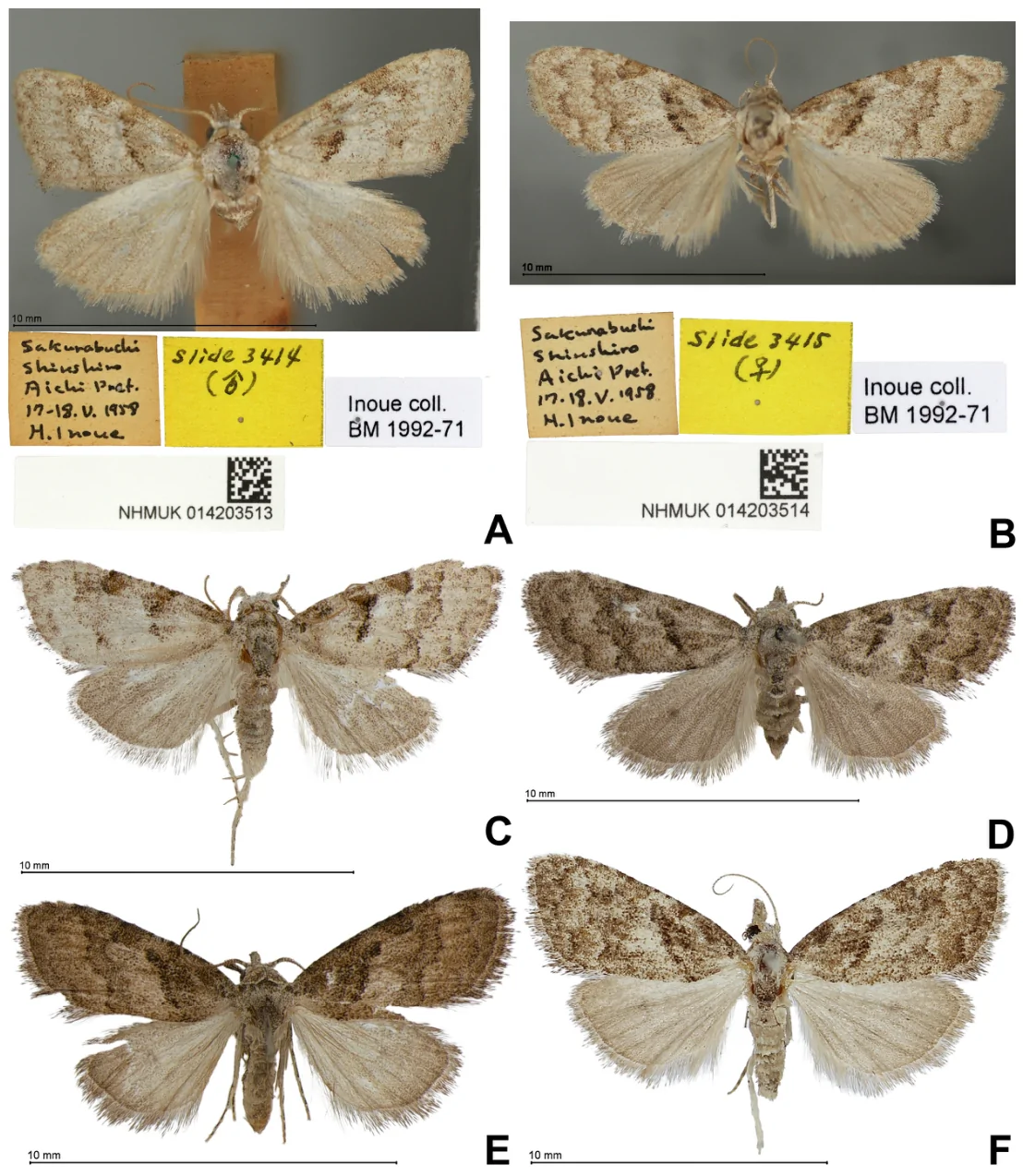
Adults of *Nola abscondita* Cha **sp. nov.** from Japan and Taiwan. (**A**) Paratype, Japan, male, NHMUK 014203513, Inoue gen. slide No. 3414, in coll. NHMUK; (**B**) Paratype, Japan, female, NHMUK 014203514, Inoue gen. slide No. 3415, in coll. NHMUK; (**C**) Paratype, Japan, male, gen. slide No. INU-12189, in coll. Y.B. Cha; (**D**) Paratype, Japan, female, gen. slide No. INU-12201, in coll. Y.B. Cha; (**E**) Paratype, Taiwan, male, gen. slide No. INU-12210, in coll. Y.B. Cha; (**F**) Paratype, Taiwan, female, NHMUK 016480176, gen. slide No. NHMUK 014334333, in coll. NHMUK.

**Description.** *Adults* (Figure 3 and Figure 4). Length of forewing 7–8 mm in male, 6–9 mm in female, wingspan 13–20 mm. Antenna bipectinate in male, filiform in female. Head and thorax brownish-grey mixed with fuscous scales. Ground color of forewing brownish-grey; elongate baso-costal patch red-brown; antemedial line black with dark ochreous highlight; medio-costal patch red-brown, semi-circular; postmedial line thin, diffuse, slightly wavy; subterminal line shadow-like, sinuous. Ground color of hindwing pale brownish-grey, with small, rounded discal dot. Abdomen dark grey.

**Figure 5:**
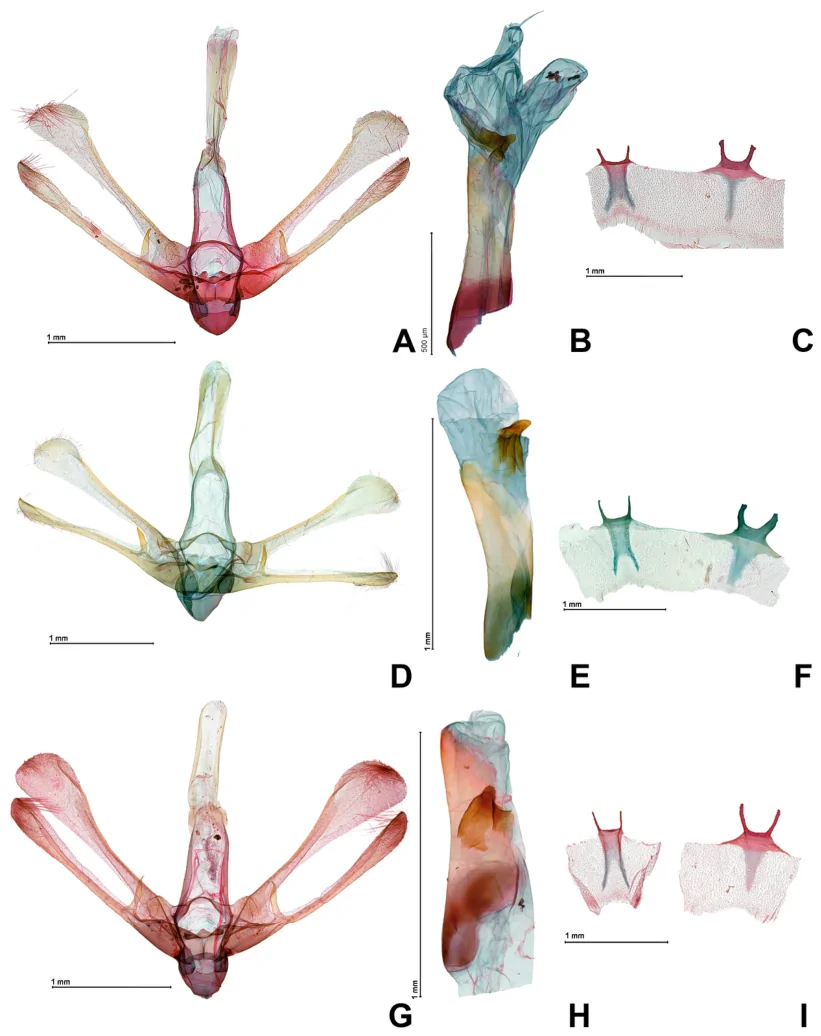
Male genitalia of *Nola abscondita* Cha **sp. nov.** from Korea (all in coll. Y.B. Cha). (**A**–**C**) holotype, gen. slide No. INU-11834; (**D**–**F**) paratype, gen. slide No. INU-11627; (**G**–**I**) paratype, gen. slide No. INU-12314. (**A**,**D**,**G**) genital capsule; (**B**,**E**,**H**) aedeagus; (**C**,**F**,**I**) Eighth segment (right: tergite, left: sternite).

**Figure 6:**
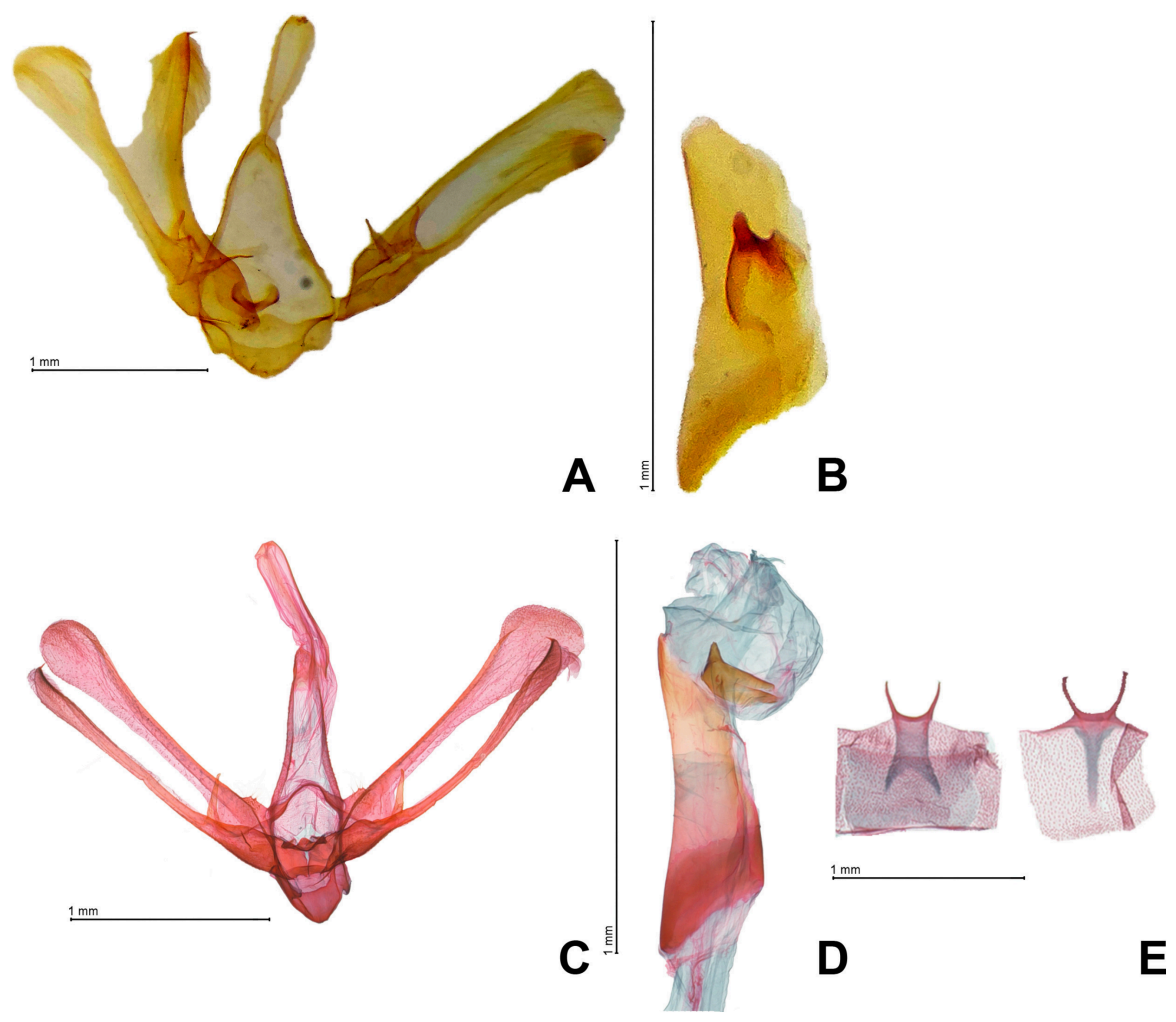
Male genitalia of *Nola abscondita* Cha **sp. nov.** from Japan. (**A**,**B**) paratype, NHMUK 014203513, Inoue gen. slide No. 3414, in coll. NHMUK; (**C**–**E**) paratype, gen. slide No. INU-12189, in coll. Y.B. Cha. (**A**,**C**) genital capsule; (**B**,**D**) aedeagus; (**E**) Eighth segment (right: tergite, left: sternite).

**Figure 7:**
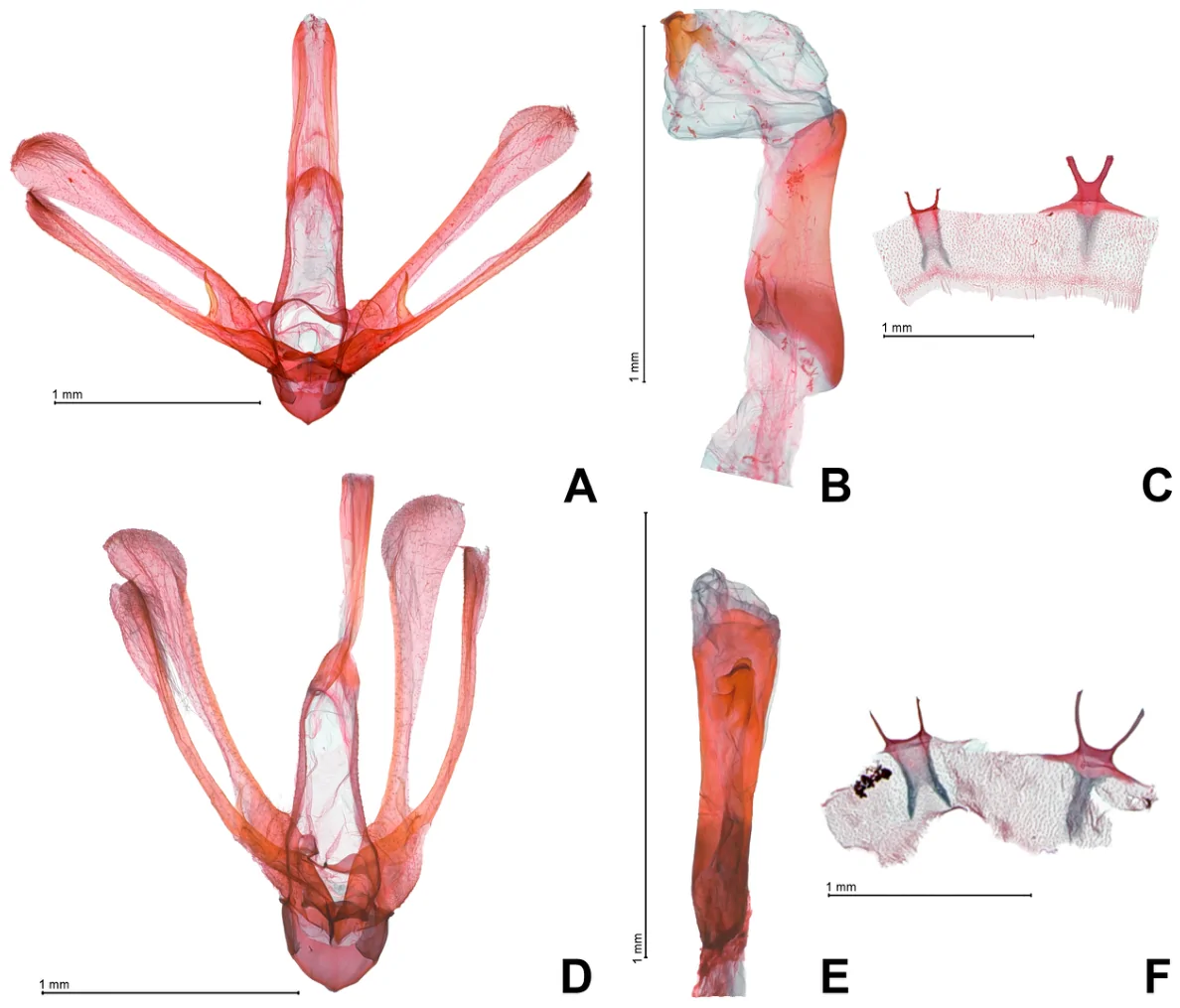
Male genitalia of *Nola abscondita* Cha **sp. nov.** from Taiwan (all in coll. Y.B. Cha). (**A**–**C**) paratype, gen. slide No. INU-12209; (**D**–**F**) paratype, gen. slide No. INU-12210. (**A**,**D**) genital capsule; (**B**,**E**) aedeagus; (**C**,**F**) Eighth segment (right: tergite, left: sternite).

*Male genitalia* (Figure 5, Figure 6 and Figure 7). Uncus entirely reduced, subscaphium with a pair of weakly sclerotized narrow longitudinal streaks. Tegumen elongate, narrow. Transtillae weakly sclerotized, basally stout, fused medially. Dorsal lobe of valva spatulate with narrow medial section; costal margin weakly sclerotized, almost straight; ventral lobe of valva very narrow, ca. 10% shorter than dorsal lobe, ventral half sclerotized, distal section slightly dilated, apex pointed bearing a short, acute spine. Harpe relatively long and narrow, almost straight, distal quarter produced into slim, apically slightly hooked process. Sacculus short and narrow. Juxta small, very short U-shaped with short rounded posterolateral projections. Vinculum short, U-shaped. Aedeagus short and moderately thick, almost straight, caecum short and rounded, carina unmodified. Vesica with a basally robust, distally tapering, apically rounded and finely dentate sub-triangular cornutus. Eighth tergite with two well-developed anterior apodemes, two stout lateral projections and a rather long, narrow, digitiform posteromedial process. Eighth sternite elongate quadrangular with two narrow anterior apodemes and two posterior processes.

**Figure 8:**
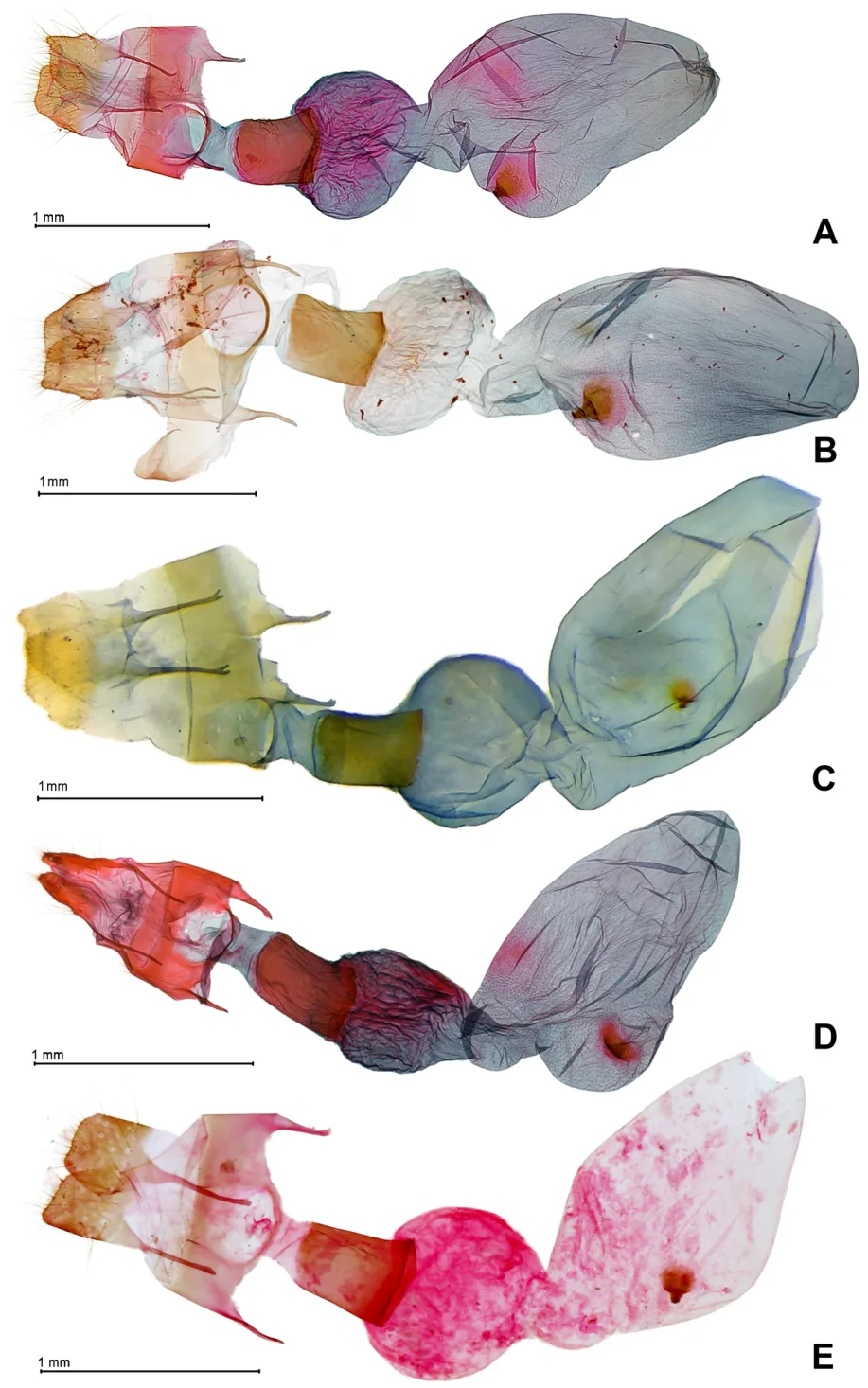
Female genitalia of *Nola abscondita* Cha **sp. nov.** from East Asia. (**A**) paratype, Korea, gen. slide No. INU-11624, in coll. Y.B. Cha; (**B**) paratype, Korea, gen. slide No. INU-12318, in coll. Y.B. Cha; (**C**) paratype, Japan, NHMUK 014203514, Inoue gen. slide No. 3415, in coll. NHMUK; (**D**) paratype, Japan, gen. slide No. INU-12201, in coll. Y.B. Cha; (**E**) paratype, Taiwan, NHMUK 016480176, gen. slide No. NHMUK 014334333, in coll. NHMUK.

*Female genitalia* (Figure 8). Apophysis posterioris thin, pointed, ca. two times longer than apophysis anterioris. Ostium bursae rather large, circular with narrow sclerotized anterior margin. Ductus bursae short, with a very short, membranous distal portion and a ca. 5 times longer, heavily sclerotized proximal portion. Cervix bursae largely inflated, rugose. Corpus bursae elongate ovoid; signum bursae with extensive, rounded well-sclerotized base continued in a short and narrow trapezoidal thorn-shaped process; in addition, other side of bursa with a small, very weakly sclerotized rounded area. Appendix bursae absent.

**Distribution.** Korea, Japan, China (Taiwan) [9,11,20,22,23].

**Remark.** *Nola yoshinensis* was reported from Korea by Oh [11] following the misidentification of the species in Inoue [9]. Recently, it has been treated as *N*. *kanshireiensis* by Sasaki [22] and Kim et al. [23]; however, genitalia illustration were not included in these papers. In this present study, the identities of *N. yoshinensis* and *N. kanshireiensis* are clarified resulting in the discovery of a long-overlooked, undescribed species.

**Etymology.** The specific epithet is derived from the Latin adjective ‘*absconditus*’, meaning ‘hidden’ or ‘concealed’. This name refers to the fact that the new species has long been ‘hidden’ under the names of *Nola yoshinensis* or *Nola kanshireiensis* due to their misidentifications.

### 3.2. Supplementary Distributional Data Sourced from the Citizen Science Platform iNaturalist

Although the identification of Nolinae species often requires examination of genitalia structures, the species treated in this paper can be reliably identified from external habitus, except in the case of heavily worn specimens. Nevertheless, the identification of additional photographic records accessed via iNaturalist remains somewhat uncertain, as these observations cannot be formally verified without examination of physical specimens. Accordingly, these records are presented separately as supplementary data. Despite these limitations, the records are sufficient to suggest that the actual distribution range of this species is broader than that inferred solely from the formally verified material.

#### 3.2.1. *Nola kanshireiensis* (Wileman & South, 1916)

iNaturalist distributional map: https://www.inaturalist.org/observations?subview=map&taxon_id=1151861 (Accessed: 8 June 2026)

**Distribution.** China (Taiwan (Taipei), Hong Kong), the Philippines (Palawan), Vietnam.

**Remark.** A total of 74 observations by 23 individual observers were reviewed. Notably, these records included single extralimital occurrences from Vietnam (with obscured geoprivacy) and the Philippines (Palawan Island). Because these specific observations fall well outside the previously established range of the species, they strongly suggest that the geographical distribution of *N. kanshireiensis* is significantly wider than historically recognized. Observation details are comprehensively listed in Table A1.

##### 3.2.1.1. *Nola sakishimana* Inoue, 2001, **syn. nov.**

iNaturalist distributional map: none

##### 3.2.1.2. *Nola spinivesica* Holloway, 2003, **syn. nov.**

iNaturalist distributional map: https://www.inaturalist.org/observations?subview=map&taxon_id=869395 (Accessed: 8 June 2026)

**Distribution.** Malaysia.

**Remark.** Only a single observation was reviewed. However, because the photograph depicts a captive individual, the recorded coordinates may not represent the precise natural locality of the species. Observation details are comprehensively listed in Table A1.

#### 3.2.2. *Nola yoshinensis* (Wileman & West, 1929)

iNaturalist distributional map: https://www.inaturalist.org/observations?subview=map&taxon_id=1308169 (Accessed: 8 June 2026)

**Distribution.** Korea.

**Remark.** A total of three observations by two observers were reviewed. All observations were made in Korea. Observation details are comprehensively listed in Table A1.

##### 3.2.2.1. *Nola ebatoi* (Inoue, 1970), **syn. nov.**

iNaturalist distributional map: https://www.inaturalist.org/observations?subview=map&taxon_id=1239408 (Accessed: 8 June 2026)

**Distribution.** Japan.

**Remark.** A total of six observations by four observers were reviewed. All individuals were recorded in Japan. Observation details are comprehensively listed in Table A1.

## 4. Discussion

*Celama yoshinensis* (Wileman & West, 1929) was originally described from Japan. Examination of the type material in the present study confirms the identity of the true *Nola yoshinensis* and demonstrates that the species concept applied by Inoue (1982) encompassed specimens that do not correspond to the true taxon (Figure 3A,B, Figure 5A,B and Figure 7C; see also Inoue [9]: 663; pl. 154, figure 25; pl. 349, figure 15; pl. 352, figure 10). This erroneous concept was subsequently adopted in several regional works, resulting in the misidentification of an undescribed species (*Nola abscondita* Cha **sp. nov.**) as *N. yoshinensis* sensu Inoue in the Korean literature [11,20].

Sasaki [22] later reinterpreted *N. yoshinensis* sensu Inoue as a synonym of *Nola kanshireiensis* (Wileman & South, 1916), thereby extending the distribution of *N. kanshireiensis* to Japan and Korea. However, this reassignment lacked explicit morphological justification and did not involve comparison with type material. The expanded but erroneous species concept was subsequently followed in later checklists, including the Korean Noctuoidea checklist by Kim et al. [23], where *N. kanshireiensis* was listed with reference to *N. yoshinensis* sensu Inoue.

Further complicating the nomenclatural confusion, *Nola sakishimana* Inoue, 2001 **syn. nov.** and *Nola spinivesica* Holloway, 2003 **syn. nov.** are conspecific with *N. kanshireiensis,* whereas *Nola ebatoi* (Inoue, 1970) **syn. nov.** is conspecific with *N. yoshinensis*.

Within the Korean fauna, all records attributed to *N. yoshinensis* sensu Inoue and *N. kanshireiensis* sensu Sasaki are demonstrated here to represent *Nola abscondita* Cha **sp. nov.** Consequently, according to our current knowledge, neither *N. yoshinensis* nor *N. kanshireiensis* occurs in Korea. Although Cha et al. [52] omitted both species from the most recent checklist of Korean Nolidae, they did not clarify their taxonomic status.

Our results show that *N. abscondita* Cha **sp. nov.** is distributed in Korea, Japan, and Taiwan. In Japan, it occurs sympatrically with *N. yoshinensis*, whereas in Taiwan it occurs sympatrically with *N. kanshireiensis*. The apparent overlap in distribution reported by Sasaki [22] therefore reflects an erroneous species concept rather than misidentification of Taiwanese material. Comparison with Hong Kong specimens of *N. kanshireiensis* illustrated by László and Sterling [46] further confirms that material previously identified as *N. kanshireiensis* from Korea and Japan does not correspond to the true species.

In this study, species delimitation was based primarily on rigorous morphological examination of primary type specimens to resolve historical nomenclatural confusion. While molecular analyses such as DNA barcoding were not performed in the present work due to the limitations of historical type material and practical constraints, future molecular studies incorporating fresh material will be essential to further test genetic divergence and cryptic diversity within this group.

## 5. Conclusions

Through re-examination of primary type material and detailed comparative morphological analyses, this study resolves the long-standing taxonomic confusion surrounding *Nola yoshinensis* and *N. kanshireiensis* in East Asia. Our results demonstrate that *Nola abscondita* Cha **sp. nov.** represents a distinct and previously unrecognized species distributed across Korea, Japan, and Taiwan. In contrast, *N. kanshireiensis* is confirmed to be restricted to Taiwan and Hong Kong, while *N. yoshinensis* is shown to occur exclusively in Japan. These findings underscore the critical importance of type-based reassessment for uncovering cryptic diversity and refining species distributions within East Asian *Nola*.

## Data Availability

The original contributions presented in this study are included in the article. Upon request to the respective repository, the specimens may be examined or made available subject to the policies and approval of each institution.

## Abbreviations

The following abbreviations are used in this manuscript:

collection depositories	
INU	Incheon National University, Incheon, Republic of Korea
KNAE	Korea National Arboretum, Pocheon, Republic of Korea
MWM/ZSM	Museum Witt in the Zoological State Collection, Munich, Germany
NHMUK	Natural History Museum, London, United Kingdom
Korean provinces	
CB	Chungchungbuk-do
CN	Chungchungnam-do
GB	Gyeongsangbuk-do
GG	Gyeonggi-do
GN	Gyeongsangnam-do
GW	Gangwon state
JB	Jeonbuk state
JN	Jeollanam-do

## Appendix A

**Table A1:** Additional material retrieved from iNaturalist. (Erroneous names applied in iNaturalist are indicated in square brackets ‘[…]’). All URLs accessed on 8 June 2026.

No.	Species	Country	URL	Geoprivacy
1	*Nola kanshireiensis*	Hong Kong	https://www.inaturalist.org/observations/251353605	Disclosed
2	*Nola kanshireiensis*	Hong Kong	https://www.inaturalist.org/observations/5249200	Disclosed
3	*Nola kanshireiensis*	Hong Kong	https://www.inaturalist.org/observations/5577429	Disclosed
4	*Nola kanshireiensis*	Hong Kong	https://www.inaturalist.org/observations/325316610	Disclosed
5	*Nola kanshireiensis*	Hong Kong	https://www.inaturalist.org/observations/326946513	Disclosed
6	*Nola kanshireiensis*	Hong Kong	https://www.inaturalist.org/observations/348498957	Disclosed
7	*Nola kanshireiensis*	Hong Kong	https://www.inaturalist.org/observations/361601619	Disclosed
8	*Nola kanshireiensis*	Hong Kong	https://www.inaturalist.org/observations/21696069	Disclosed
9	*Nola kanshireiensis*	Hong Kong	https://www.inaturalist.org/observations/64086852	Disclosed
10	*Nola kanshireiensis*	Hong Kong	https://www.inaturalist.org/observations/101249555	Disclosed
11	*Nola kanshireiensis*	Hong Kong	https://www.inaturalist.org/observations/101364653	Disclosed
12	*Nola kanshireiensis*	Hong Kong	https://www.inaturalist.org/observations/9561137	Disclosed
13	*Nola kanshireiensis*	Hong Kong	https://www.inaturalist.org/observations/92724056	Disclosed
14	*Nola kanshireiensis*	Hong Kong	https://www.inaturalist.org/observations/203564660	Disclosed
15	*Nola kanshireiensis*	Hong Kong	https://www.inaturalist.org/observations/149467972	Disclosed
16	*Nola kanshireiensis*	Hong Kong	https://www.inaturalist.org/observations/100657415	Disclosed
17	*Nola kanshireiensis*	Hong Kong	https://www.inaturalist.org/observations/194037215	Disclosed
18	*Nola kanshireiensis*	Hong Kong	https://www.inaturalist.org/observations/154095255	Disclosed
19	*Nola kanshireiensis*	Hong Kong	https://www.inaturalist.org/observations/264937269	Disclosed
20	*Nola kanshireiensis*	Hong Kong	https://www.inaturalist.org/observations/80455678	Disclosed
21	*Nola kanshireiensis*	Hong Kong	https://www.inaturalist.org/observations/193922070	Disclosed
22	*Nola kanshireiensis*	Hong Kong	https://www.inaturalist.org/observations/356099808	Disclosed
23	*Nola kanshireiensis*	Hong Kong	https://www.inaturalist.org/observations/136569889	Disclosed
24	*Nola kanshireiensis*	Hong Kong	http://www.inaturalist.org/observations/1459598	Disclosed
25	*Nola kanshireiensis*	Hong Kong	https://www.inaturalist.org/observations/191250285	Disclosed
26	*Nola kanshireiensis*	Hong Kong	https://www.inaturalist.org/observations/194453427	Disclosed
27	*Nola kanshireiensis*	Hong Kong	https://www.inaturalist.org/observations/236586794	Disclosed
28	*Nola kanshireiensis*	Hong Kong	https://www.inaturalist.org/observations/301502626	Disclosed
29	*Nola kanshireiensis*	Hong Kong	https://www.inaturalist.org/observations/321174193	Disclosed
30	*Nola kanshireiensis*	Hong Kong	https://www.inaturalist.org/observations/90889658	Disclosed
31	*Nola kanshireiensis*	Hong Kong	https://www.inaturalist.org/observations/247320390	Disclosed
32	*Nola kanshireiensis*	Hong Kong	https://www.inaturalist.org/observations/191334245	Disclosed
33	*Nola kanshireiensis*	Hong Kong	https://www.inaturalist.org/observations/193850181	Disclosed
34	*Nola kanshireiensis*	Hong Kong	http://www.inaturalist.org/observations/1912228	Disclosed
35	*Nola kanshireiensis*	Hong Kong	https://www.inaturalist.org/observations/118757726	Obscured
36	*Nola kanshireiensis*	Hong Kong	https://www.inaturalist.org/observations/162072174	Obscured
37	*Nola kanshireiensis*	Hong Kong	https://www.inaturalist.org/observations/192185700	Obscured
38	*Nola kanshireiensis*	Hong Kong	https://www.inaturalist.org/observations/192185719	Obscured
39	*Nola kanshireiensis*	Hong Kong	https://www.inaturalist.org/observations/204161763	Obscured
40	*Nola kanshireiensis*	Hong Kong	https://www.inaturalist.org/observations/204375918	Obscured
41	*Nola kanshireiensis*	Hong Kong	https://www.inaturalist.org/observations/207894350	Obscured
42	*Nola kanshireiensis*	Hong Kong	https://www.inaturalist.org/observations/210904374	Obscured
43	*Nola kanshireiensis*	Hong Kong	https://www.inaturalist.org/observations/213253709	Obscured
44	*Nola kanshireiensis*	Hong Kong	https://www.inaturalist.org/observations/214180973	Obscured
45	*Nola kanshireiensis*	Hong Kong	https://www.inaturalist.org/observations/215237511	Obscured
46	*Nola kanshireiensis*	Hong Kong	https://www.inaturalist.org/observations/227231281	Obscured
47	*Nola kanshireiensis*	Hong Kong	https://www.inaturalist.org/observations/230223552	Obscured
48	*Nola kanshireiensis*	Hong Kong	https://www.inaturalist.org/observations/238912811	Obscured
49	*Nola kanshireiensis*	Hong Kong	https://www.inaturalist.org/observations/244128093	Obscured
50	*Nola kanshireiensis*	Hong Kong	https://www.inaturalist.org/observations/251510375	Obscured
51	*Nola kanshireiensis*	Hong Kong	https://www.inaturalist.org/observations/252838220	Obscured
52	*Nola kanshireiensis*	Hong Kong	https://www.inaturalist.org/observations/262951769	Obscured
53	*Nola kanshireiensis*	Hong Kong	https://www.inaturalist.org/observations/268888666	Obscured
54	*Nola kanshireiensis*	Hong Kong	https://www.inaturalist.org/observations/277286242	Obscured
55	*Nola kanshireiensis*	Hong Kong	https://www.inaturalist.org/observations/295070797	Obscured
56	*Nola kanshireiensis*	Hong Kong	https://www.inaturalist.org/observations/316296861	Obscured
57	*Nola kanshireiensis*	Hong Kong	https://www.inaturalist.org/observations/321247685	Obscured
58	*Nola kanshireiensis*	Hong Kong	https://www.inaturalist.org/observations/321767461	Obscured
59	*Nola kanshireiensis*	Hong Kong	https://www.inaturalist.org/observations/324442325	Obscured
60	*Nola kanshireiensis*	Hong Kong	https://www.inaturalist.org/observations/325462644	Obscured
61	*Nola kanshireiensis*	Hong Kong	https://www.inaturalist.org/observations/326000632	Obscured
62	*Nola kanshireiensis*	Hong Kong	https://www.inaturalist.org/observations/327095138	Obscured
63	*Nola kanshireiensis*	Hong Kong	https://www.inaturalist.org/observations/327224588	Obscured
64	*Nola kanshireiensis*	Hong Kong	https://www.inaturalist.org/observations/330692225	Obscured
65	*Nola kanshireiensis*	Hong Kong	https://www.inaturalist.org/observations/331284640	Obscured
66	*Nola kanshireiensis*	Hong Kong	https://www.inaturalist.org/observations/336469542	Obscured
67	*Nola kanshireiensis*	Hong Kong	https://www.inaturalist.org/observations/337662463	Obscured
68	*Nola kanshireiensis*	Hong Kong	https://www.inaturalist.org/observations/337671434	Obscured
69	*Nola kanshireiensis*	Hong Kong	https://www.inaturalist.org/observations/348428231	Obscured
70	*Nola kanshireiensis*	Hong Kong	https://www.inaturalist.org/observations/350691522	Obscured
71	*Nola kanshireiensis*	Hong Kong	https://www.inaturalist.org/observations/357858614	Obscured
72	*Nola kanshireiensis*	Taiwan	https://www.inaturalist.org/observations/271855935	Obscured
73	*Nola kanshireiensis*	Vietnam	https://www.inaturalist.org/observations/36152450	Obscured
74	*Nola kanshireiensis*	the Philippines	https://www.inaturalist.org/observations/334630071	Disclosed
75	*Nola kanshireiensis*, as [*Nola spinivesica*]	Malaysia	https://www.inaturalist.org/observations/165295402	Disclosed
76	*Nola yoshinensis*, as [*Nola ebatoi*]	Japan	https://www.inaturalist.org/observations/351456512	Disclosed
77	*Nola yoshinensis*, as [*Nola ebatoi*]	Japan	https://www.inaturalist.org/observations/208689079	Disclosed
78	*Nola yoshinensis*, as [*Nola ebatoi*]	Japan	https://www.inaturalist.org/observations/147350268	Disclosed
79	*Nola yoshinensis*, as [*Nola ebatoi*]	Japan	https://www.inaturalist.org/observations/138098168	Disclosed
80	*Nola yoshinensis*, as [*Nola ebatoi*]	Japan	https://www.inaturalist.org/observations/136267080	Disclosed
81	*Nola abscondita* **sp. nov.** as [*Nola yoshinensis*]	Japan	https://www.inaturalist.org/observations/107023265	Disclosed
82	*Nola yoshinensis*, as [*Nola ebatoi*]	Korea	https://www.inaturalist.org/observations/314979321	Disclosed
83	*Nola abscondita* **sp. nov.** as [*Nola yoshinensis*]	Korea	https://www.inaturalist.org/observations/314979261	Disclosed
84	*Nola abscondita* **sp. nov.** as [*Nola yoshinensis*]	Korea	https://www.inaturalist.org/observations/285892736	Disclosed
